# Electromechanical Impedance Data-Driven Metal Structural Tensile Stress Identification Using Generative Adversarial Networks

**DOI:** 10.3390/ma19122445

**Published:** 2026-06-08

**Authors:** Demi Ai, Rui Zhang

**Affiliations:** School of Civil and Hydraulic Engineering, Huazhong University of Science and Technology, 1037 Luoyu Road, Wuhan 430074, China

**Keywords:** tensile stress estimation, electromechanical admittance (EMA), piezoelectric ceramic (PZT), EMA generative adversarial network (EMAGAN), data enhancement, deep learning

## Abstract

Deep learning networks facilitate automated metal material/structural stress identification when employing the electromechanical impedance/admittance (EMI/EMA) of piezoelectric ceramic (PZT) transducers, while insufficient data quantity and low quality usually restrict the performance of data-driven deep networks. To address this problem, this paper innovatively proposed an original data enhancement method using the EMA generative adversarial network (EMAGAN) to overcome measurement data inefficiency and deficiency for deep learning-based stress identification, which is difficult to accomplish using the traditional EMA technique. In this method, a novel data-normalized algorithm was tuned to collaboratively foster the EMAGAN-based dataset generation. Then, the synthetic datasets incorporated with original ones were fed into an adaptively established one-dimensional convolutional neural network (1DCNN) for accurate stress prediction. A validating experiment was performed on an aluminum beam specimen subjected to uniaxial tensile load until failure, which was continuously monitored via two surface-bonded PZT transducers. The efficacy of the generated EMA datasets was evaluated through comparison with the raw ones in terms of statistical errors and deep learning-based aluminum structural stress identification. The results demonstrated that the EMAGAN generated high-accuracy EMA data which exceeded 380 times that of the normal collection method, and the EMAGAN paired with 1DCNN provides a promising way for EMA data-driven metal structural stress identification with high efficiency, intelligence and accuracy.

## 1. Introduction

Accurate identification of in situ stress variations in metal materials or structural components, such as aluminum/steel beams, slabs and columns, holds the key to diagnosing the source of initiated abnormity/damage in the structural health monitoring (SHM) domain; it is also important in evaluating metal structural safety, assessing remaining structural life and determining a maintenance schedule [1,2,3]. Traditional and recently developed SHM techniques have promoted the detection of stress levels in structural components. For example, the hole-drilling method using a strain gage is one of the most widely employed approaches to determine near-surface stresses [4]. The X-ray or neutron diffraction method can estimate the stresses of structural components by measuring the lattice spacing of materials [5]. However, the hole-drilling method is destructive when drilling a hole in a structure, and X-ray measurements require expensive facilities; neither are suitable for continuous monitoring of large operating structures. Other nondestructive methods such as the stress function method can estimate the residual stress of engineering structures, while requisite resolution of complex mathematical problems, like the nonlinear optimization of structural models, is required upon solving [6]. The digital image correlation technique is non-contact and estimates a stress field by computing the correlation of images obtained from unstressed and stressed conditions [7]; however, it requires a speckle pattern on the structural surface, thus being tedious and having poor efficiency for continuous monitoring. In addition, the ultrasonic technique mainly based on acoustoelastic effects can realize nondestructive and continuous stress measurements [8,9,10]; unfortunately, the high requirements for the geometric structure of the surface make it only suitable for detecting simple structural elements of steel and aluminum, and the high cost and bulky equipment also hinder its extensive applications [11].

Different from the above-mentioned approaches, the electromechanical admittance (inverse of impedance, EMA/EMI) technique only needs one piezoelectric ceramic (PZT) patch as an actuator/sensor and has shown preferable merits, including fast responses, low cost, non-intrusiveness, and online monitoring ability, over the past three decades [12]. The operation of the technique involves bonding the PZT to a component or structural element, and the PZT sheet is actuated by a sinusoidal voltage generated via a commercial impedance analyzer, which transmits the responding vibrations to the component and leads to its deformation. Meanwhile, the PZT behaving as a sensor obtains the deformation response from the component and outputs an electric signal, i.e., the EMA signatures. Many studies have applied the impedance technique to the detection of defects such as cracks, local-bolt looseness, reinforcement corrosion and stiffness degradation in metal, composite and concrete structures [13,14,15,16,17]. Owing to its ultrasonic-frequency operations, it is effective for damage identification at incipient levels and without interference of normal operating conditions, vibrations, or boundary changes in the target structure, which also enables it to monitor load/stress-induced damages. The feasibility of the EMI-based method was validated in the detection and characterization of the propagation of fatigue cracks and remaining useful life in an aluminum beam specimen subjected to cyclic tensile load [18]. Piezo-based configurations were comparatively applied to detect impact load-induced damage with root mean square deviation (RMSD) variation, which could discern imminent failure of an RC slab [19]. In addition, voltage responses, the band width of resonant peaks and RMSD index values of piezoelectric transducers provided cogent evidence of shear-load damage, localized cracks and the prediction of the forthcoming final shear failure at the earlier onset of diagonal cracking stages [20,21,22].

Focusing on load monitoring via the EMA technique, extensive analytical and experimental investigations have been conducted to reveal the characteristics of the EMA signature responding to static/dynamic loads. Experiments on laboratory-sized aluminum beam specimens demonstrated that vertical and lateral shifts in the peaks in the conductance signatures were induced by transverse loading, lateral movements like anticlockwise rotation, and splitting/merging of peaks, and emerged new peaks were more prominent in susceptance signals [23]. The conductance signatures of encapsulated PZT inside a concrete structure demonstrated higher sensitivity to the applied load that is vertical to the planar direction of the sensor, while showing sensitivity to structural damage variations when vertical to the thickness direction of the sensor [24]. Tests showed that the applied load also led to an increment in the amplitude and a rightward shift in the EMA spectrum, thus behaving as indicative of the damage/stress state of substrate in terms of the strain level and the extent of damage [25,26]. In addition to these, experiments on a lab-scale metal beam subjected to axial load indicated that the load was more influential to the transverse mode of the structure, and the boundary conditions were found to be critical in causing a local stiffening effect, thus overwhelming the softening effect derived from compression [27]. Based on those features, it is possible to monitor load stress on structures interrogated with PZT transducers. Depending on the Lagrangian approach and direct piezoelectric effect, a flexional piezoelectric force sensor was proposed to detect dynamic/static force [28]. Another portable PZT-interface sensing technology was developed for cable force-loss detection in an anchorage structure [29]. Load monitoring tests on a steel gear also indicated that the frequency responses ranging from 50 to 60 kHz were enough for load detection along the thickness direction of the PZT transducer [30]. Other load-sensing methods using different types of PZT sensors mainly employed the dominating resonant peak of PZT resistance from cable-deck-connection devices [31] or a bolted connection for detecting pre-load looseness [32]. The frequency-domain EMA signatures were also transformed into time-domain ones for strain sensing by wire/wireless sensor networks during cycles of loading/unloading [33]. In addition, for load detection along the transverse and perpendicular direction to the plane of the engineering structure, researchers developed ultra-sensitive contactless monitoring methods [34]. From those studies, the susceptance signals were found as a preferable indicator for detecting transversely loaded structures, and conversely the conductance signals were found more useful for detecting axially loaded structures or monitoring perpendicular loads on the target structure [35,36].

Specifically, in the matter of precise evaluation of the in situ stresses in concrete or metal structures, traditional studies have been conducted to build the internally functional relationships between the PZT-coupled impedance models and structural stress levels. For example, the theory of Euler–Bernoulli beam was employed to derive the dynamic responses of an axially loading beam coupled with two bonded PZT transducers [37]. Similarly, the beam model derived to compute the point-wise dynamic stiffness of the structure indicated that linear shifts of the resonance peaks of conductance signals were effective indicators of the uniaxial stress increment applied on a metal beam under decreased loads from axial tension to compression [38]. Rapid monitoring of applied loads by faster impedance spectrums was acquired by eliminating unimportant resonant peaks in the measured EMA signals [39]. To resolve the problem of the closed-form point-wise dynamic stiffness that was restricted by setting certain structural models, the impedance theory of new mechanical impedance was introduced to realize the simultaneous detection of tension and compression stress in a flexure-critical beam structure [40]. An impedance analysis model of an embedded piezoelectric plate was also proposed to account for static stress detection based on nonlinear elastic dynamic governing equations and the displacement method [41]. Flexure-critical tensile and compressive stress detection in concrete structures was realized by formulating a 2D embedded PZT structural mechanical impedance interaction model [42]. Hierarchical clustering analysis was also employed to analyze the EMI signature to identify load-induced damage in fiber-reinforced concrete cylinders subjected to repeated loading [43]. Despite that these models could calculably interpret structural stress in a measurable way, a few issues remain challenging: (1) they required comprehensive knowledge about the mechanism of the EMI model, as well as the values of all constants involved with the structure, adhesive and PZT materials [44]; (2) such evaluation criteria is essentially experiential-based prescription and qualitative, as the EMA variations could usually be complicated, irregular or even contradictory when subjected to the tensile and compressive stress [23,27]; (3) manual signal analysis or processing could be significantly time-consuming and lowly efficient, making it unreliable for dealing with considerable signals in long-term monitoring.

Recently, owing to the booming progress of computer vision and machine learning operations, new breakthroughs have been ushered in in the SHM domain using the EMA technique, and new approaches have also been created for structural stress identification, especially in automated feature extraction. Data compression via principal component analysis and k-means-clustering-based pattern recognition were employed to enhance the capability of raw EMI/EMA data analysis by maintaining the critically essential features, meanwhile decreasing the unwanted noises by performing data compression [45,46]. Neural networks were employed for learning EMA features for structural damage detection, which were capable of autonomously identifying damage-sensitive ranges of frequency and outputting more accurate results like the damage type, severity or location [47,48,49,50]. Differing from traditional machine learning approaches, deep neural networks have shown overwhelming excellence in the field of computer vision, among which the convolutional neural network (CNN) has been regarded as a state-of-art approach for object classification and detection not only in accuracy but also in speed [51]. Recent investigations have demonstrated the implementation of CNN-based deep learning for damage detection in the fault diagnosis of motor conditions [52], vibration-based damage detection [53], crack identification of concrete/RC structures [54], and bearing fault diagnosis [55]. With regard to the integration of the EMA technique, CNNs have also been applied to classify structural integrity under varied temperature conditions [56] to identify damages on an aluminum plate structure by simulating mass-additional points [57,58]. In the studies conducted by the authors of [59,60], one-dimensional (1D) and two-dimensional (2D) CNNs were proposed, respectively, for learning graphs from the Pearson correlation coefficient and raw EMA signals for the detection of artificial damages under varied temperatures or compressive stress and compression-induced damages in concrete cubic structures.

Although those deep learning approaches achieve high accuracy in the automated identification of damages or stress levels, their major limitation is that they require sufficiently large and effective EMA datasets to train and test the deep CNN models. In the previous model, 900 groups of EMA-signal-derived root mean square propagation were obtained to train a 5-layer 1D CNN model [56]. The other CNN models were also fed by 1080 groups of signals [57], 1260 groups [58], 804 groups [59] and 520 datasets of the EMA-derived or the raw conductance spectra [60] as well. Recently, deep learning models were also developed for the quantitative prediction of torque values in bolted joints of steel truss structures via collecting 528 impedance signatures [61], impact detection, and assessment of the internal damage of concrete structures using 480 samples [62]. An efficient automatic detection method for bolt loosening in steel frame structures was also achieved by integrating a wearable lightweight impedance system and a deep learning model that fused multi-source information [63]. 2D CNN models were applied to learn the EMI responses of a capsule-like smart aggregate, and the results demonstrated its potential for estimating stress and damage in concrete structures via collecting 240 signals for each stress level [64]. A domain adaptation technique was implemented for the axial stress evaluation of plate rubber bearings with deep learning of the EMA signals via a novel residual block-domain adaptation neural network [65]. Deep learning combined with the EMA technique was successfully introduced to diagnose prestress loss and steel anchorage damage, including strand damage and bearing plate damage [66,67]. However, repeatedly measuring such a considerable number of EMA signatures is not favorable for in situ stress identification, thereby hindering the practical applications of these models for engineering SHM. Perturbation of EMA responses caused by slight stress variation makes it unwarrantable to successively collect a valid EMA dataset due to its high sensitivity to stress variation [38,39,40,41,42,43,44], given that stresses in structures are usually variable, resulting in data deficiency. Moreover, continuously measuring EMA samples is laborious and inefficient even for a single stress condition, since the scanning of merely one single EMA signal may consume more than two minutes of time and for thousands of signals in total cost dozens of hours.

To overcome these limitations, this paper formulated a generative adversarial network (GAN)-based EMA data enhancement method, which is aimed at automated structural stress identification using CNN. As a lately developed and powerful network, GAN is capable of completely and adaptively excavating the underlying features or internal connections between the learning images [68]. GAN has been successfully applied to recover photo-realistic textures from heavily downsampled images [69], which was also confirmed to be effective at synthesizing photos from label maps, colorizing images and reconstructing objects from edge maps [70]. For SHM response reconstruction, GANs have typically been employed to model the mapping from the noise distributions and temporal vibrated data of real machinery; then, it generates fake datasets to balance or expand the available sets for fault diagnosis in rotating machinery [71,72,73]. Other GANs, such as balanced semisupervised GAN (BSS-GAN) and segment-based conditional generative adversarial network (SegGAN), have been proposed to address dynamic structural response reconstruction issues for spalling, crack and damage recognition [74,75]. Recently, an adapted cycle-consistent generative adversarial network (CycleGAN) that incorporated physical constraints and a self-attention mechanism was applied in the SHM field for mutual transformation of the multi-channel time series obtained from numerical models and actual structures [76]. Data augmentation was also achieved using GAN on transient, time-dependent vibration data from machine learning algorithms [77]. GAN was also implemented to recover time-series data in the context of SHM by acquiring the distribution of the original data [78] and to realize missing data imputation for the SHM of dams [79]. Unfortunately, to date, GANs have neither been applied to EMA data-based stress identification for addressing data synthesis nor been involved with metal structural stress identification issues. Actually, GAN has the capability of resolving multi-resonant EMI/EMA signatures, since the generative network and discriminative one in such a GAN model are fully involved in the process of continuous adversarial training, unless the balanced point shows that the produced data distributions have approximated to real ones. Based on the GAN, a data enhancement architecture called EMA generative adversarial network (EMAGAN) is developed in this study to composite a sufficient dataset for precise metal stress identification. The main contributions of this work are as follows:

(1) A GAN-based admittance data enhancement framework, i.e., EMAGAN, is formulated to generate qualified synthetic EMA signals for metal structures, which eliminates measuring inefficiency and deficiency with a speed hundreds of times higher than conventional measuring approaches.
(2) A novel normalization algorithm was tuned to collaboratively foster the EMAGAN-based data generation, which profitably reduces more than 22.5 times of errors for the generated data. Generated datasets feeding to the CNN model achieve higher accuracy and faster convergence for structural stress prediction.

This paper presents an introduction on the EMA and GAN approaches in the Section 2, and Section 3 presents the EMAGAN and data-enhancement algorithm for metal stress prediction. In Section 4, a proof validation is performed on the automated identification of tensile stress for an aluminum laboratory structure subjected to uniaxial loading, including a new algorithm of data normalization, training, validation and testing CNN for stress quantification. Section 5 summarizes the conclusions.

## 2. Methodology

### 2.1. EMA Technique for Stress Identification

**Figure 1:**
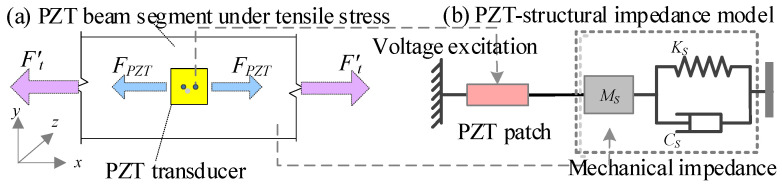
(**a**) PZT beam structural specimen under tensile stress using bonded PZT patch, (**b**) PZT structural impedance model for tensile stress detection [80].

Structural damage or stress identification using the EMA technique relies on the direct piezoelectric effect of PZT material, which generates surface charges under applied mechanical stresses. Conversely, an applied electric field induces mechanical strains through the converse piezoelectric effect. When a PZT sheet is bonded to a target component/structure being loaded, load-induced stress and damages affecting the structure would change the EMA signals, making it possible for stress/damage identification. Given that the PZT patch was connected to the structure subjected to a uniaxial load, as shown in Figure 1a, the traditional mechanism referring to the stress developed in the patch closely depends on the stiffness ratio between the structure and PZT, the PZT strain, and the shear modulus of the bonding layer [38]. To circumvent the difficulty of determining the dynamic stiffness of the structure, which involves higher-order elastic, piezoelectric and dielectric properties of the PZT along with mechanical damping effects and dielectric dissipation, a new model derived from the definition of mechanical impedance was proposed for interpreting a thin beam with a surface-bonded PZT patch being loaded with axial force [40]. In a traditional PZT structural impedance model, as shown in Figure 1b [80], the PZT is regarded as a thin bar that vibrates in the length direction and interacts with the structure replaced by its driving-point impedance, which is simplified as a single-degree-of-freedom system containing basic mechanical elements such as mass, damper, and stiffness. In Figure 1, axial-tensile-force-induced tension stress makes the beam stiffer, hence resulting in larger structural impedance; conversely, compression results in a smaller one [39,40]. Taking this effect into the expression of structural mechanical impedance, a new PZT structure interaction model to estimate structural tension/compression stress is expressed as [40]

(1) $$Y(\omega )=G(\omega )+B(\omega )j=\omega j\frac{b_{a}l_{a}}{h_{a}}\left[(\bar{\varepsilon_{33}^{T}}-d_{31}^{2}\bar{Y_{11}^{E}})+\frac{d_{31}^{2}\bar{Y_{11}^{E}}Z_{a}}{TZ_{s}+Z_{a}}\left(\frac{\tan\kappa l}{\kappa l}\right)\right]$$

where B(ω) and G(ω) denote the susceptance and conductance, respectively; *j* is the imaginary unit; ω is the angular frequency; *b_a_*, *l_a_*, and *h_a_* denote the width, length, and thickness of the PZT patch, respectively; $\bar{\varepsilon_{33}^{T}}=\varepsilon_{33}^{T}(1-\delta j)$ and $\bar{Y_{11}^{E}}=Y_{11}^{E}(1+\eta j)$ respectively denote the electric permittivity at constant stress and the complex Young’s modulus at a constant electric field; η denotes the mechanical loss factor; δ denotes the dielectric loss factor; *d*_31_ denotes the coupling piezoelectric constant; $Z_{s}$ denotes the mechanical impedance of structure and $Z_{a}$ that of PZT; and *T* denotes stress factor that is larger than one when the beam is subjected to tensile stress.

Except for Equation (1), which provides the principle of detecting structural stress, some statistical indicators like the correlation coefficient (CC), mean absolute percentage deviation (MAPD), or RMSD can compute the absolute variations in the EMA signatures between the reference and modified conditions. Many studies have verified the effectiveness of these metrics in the evaluation of damage growth, stress development and structural performance [19,20,21,22,23,24,25,26,27]. In this study, CC and RMSD indexes correlated to the changes of EMA signatures are adopted for stress evaluation, of which the expressions are denoted as

(2) $$CC=\frac{\frac{1}{N}\sum_{i=1}^{N}\left(G_{i}^{0}-\bar{G^{0}})(G_{i}^{1}-\bar{G^{1}}\right)}{\sigma_{0}\sigma_{1}}$$

(3) $$RMSD=\sqrt{\left(\frac{\sum_{i=1}^{N}(G_{i}^{1}-G_{i}^{0}{)}^{2}}{\sum_{i=1}^{N}(G_{i}^{0}{)}^{2}}\right)}$$

where $G_{i}^{0}$ and $G_{i}^{1}$ respectively denote the EMA spectrum before and after stress variation or damage at the *i*th sample point; $\bar{G^{0}}$ and $\bar{G^{1}}$ respectively denote the mean value of conductance samples before and after stress variation or damage; $\sigma_{0}$ and $\sigma_{1}$ are the corresponding standard deviations; and *N* is the sampling point of the one-time scanning measurement. While EMA characteristics or CC/RMSD metrics could provide comparative information about the relative and absolute changes in the signal measurements, they are dominantly qualitative and experience-based; thus, it is hard to provide exact information on damage severity degree or stress level [12,13,14]. Therefore, automatic feature extraction of the EMA signature with respect to a certain stress level are essential for the EMA-based SHM, particularly in practical applications. Such a drawback is expected to be overcome by utilizing deep learning of EMA datasets enhanced by GANs, as illustrated in Section 2.2.

### 2.2. Principle of GANs

In this study, automated identification of structural stress is achieved by using deep learning of raw EMI/EMA data enhanced by GAN. As an unsupervised generative deep learning model, GAN provides a solution to the problem of data imbalance [81]. GAN consists of two network modules trained in an adversarial way, namely generator (G) and discriminator (D). Figure 1 illustrates the training process of a GAN. G learns the feature distribution of the known data space and simulates the generation of new sample data. D is a binary classification network responsible for distinguishing the feature differences between real data and the data produced by G. Through adversarial training and iterative optimization, the two networks converge to a Nash equilibrium [82], where the distribution of generated data converges with that of the real data, and the discriminator can no longer effectively distinguish between them. The key to GAN training lies in the design of the loss function. During iterative adversarial training, the two network models are optimized via a maximum–minimum loss function. Given that the objectives for the discriminator *G* and the generator *D* are opposite, its objective function could be expressed:

(4) $$\underset{G}{\min}\;\underset{D}{\max}V\left(D,G\right)=\underset{G}{\min}\;\underset{D}{\max}E_{x\sim P_{r}}\left[logD\left(x\right)\right]+E_{z\sim P_{g}}[\log\left(1-D\left(G\left(z\right)\right)\right]$$

where $P_{g}$ and $P_{r}$ respectively denote the probability distribution of input noise *z* and the real sample *x*; E is a mathematical expectation; and $G\left(z\right)$ denotes the sample data from the generative network. However, despite that the GAN can produce higher-quality samples compared to alternative methods [73,74,75], the model frequently suffers from issues such as gradient dispersion and model collapse during training. Consequently, balancing the training of the generator and discriminator often demands significant effort and time. To address these challenges, a Wasserstein generative adversarial network (WGAN) is introduced [83], which employs the Wasserstein distance as a metric to quantify the divergence between the true data distribution and the generated data distribution. By utilizing this metric, the WGAN mitigates the problem of vanishing gradients on low-dimensional manifolds—a situation that arises when the generated data distribution fails to align with the real data distribution. The WGAN loss function is formulated as follows:

(5) $$L_{WGAN}=\underset{\tilde{x}∼P_{g}}{E}\left[D\left(\tilde{x}\right)\right]-\underset{x∼P_{r}}{E}\left[D\left(x\right)\right]$$

where $\tilde{x}=G(z)$. Equation (5) yields a function for which the input gradient is more stable than that of a standard GAN, thereby simplifying generator optimization [76]. However, calculating the Wasserstein distance requires enforcing a 1-Lipschitz constraint, typically attempted through weight clipping in the WGAN. This approach often leads to vanishing or exploding gradients unless the clipping threshold is meticulously tuned. To address this limitation, a gradient penalty (GP) method was introduced to directly perform the Lipschitz constraint. This produces more useful gradients that avoid vanishing or exploding, enabling the training of more complex network architectures. The resulting model, termed WGAN-GP, has a loss function formulated as follows:

(6) $$L=\underset{\tilde{x}∼P_{g}}{E}\left[D\left(\tilde{x}\right)\right]-\underset{x∼P_{r}}{E}\left[D\left(x\right)\right]+\lambda \underset{\hat{x}∼P_{\hat{x}}}{E}\left[{\left({∥\nabla_{\hat{x}}D\left(\hat{x}\right)∥}_{2}-1\right)}^{2}\right]$$

where $\hat{x}$ can be computed via the interpolation of the real sample *x* and the generated sample $\tilde{x}$ and λ is the coefficient of gradient penalty. Compared with Equation (5), the difference of the loss function for the WGAN-GP and WGAN is only a regular term added, namely, GP. Motivated by WGAN-GP, this study proposes the framework of EMAGAN, which is developed for EMA signal enhancement, as addressed in the next section.

## 3. Proposed Framework for Automated Stress Identification

### 3.1. Architecture of EMAGAN

**Figure 2:**
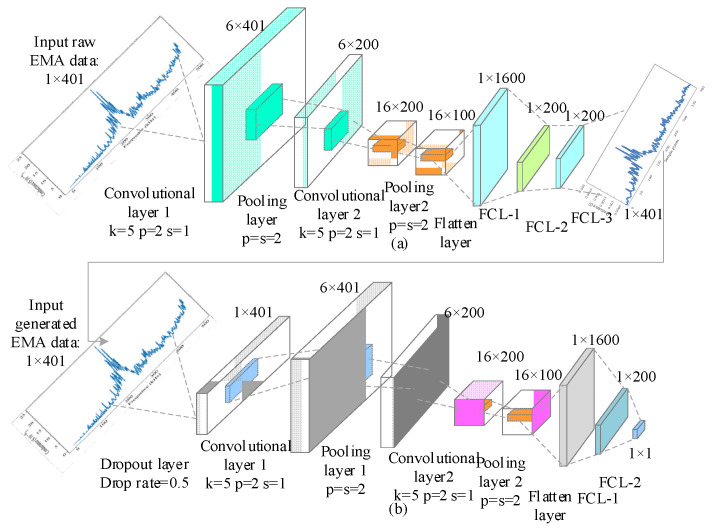
Configuration of (**a**) generator and (**b**) discriminator of the EMAGAN.

The EMAGAN was developed to produce sufficient admittance signals for the deep learning-based stress detection. As noted, several limitations exist in EMA-based SHM practice: (1) insufficient state-specific data under stress-varied conditions, (2) the high cost and low efficiency of measuring EMA datasets, and (3) identification uncertainties or low accuracy due to reliance on empirical knowledge. Therefore, sample generation and automatic detection are essential to minimize the overall cost. EMAGAN aims to achieve optimal accuracy and efficiency through artificial intelligence, which addresses the challenge of directly evaluating in situ structural stress by utilizing the complex mappings between EMA responses and stress levels, which are established through deep learning and exhibit strong correlation. The architecture of EMAGAN was determined through a trial-and-error process, including decisions on the number of hidden layers, neurons per layer, and the optimizer. Here, neurons are the basic computing units in neural networks. Neurons are referred to as nodes or units. They receive inputs from other nodes or external sources and calculate outputs. Each input has a corresponding weight, which is assigned based on the relative importance of the input information. The node provides the weighted sum of the inputs to the function. It is worth noting that configuring the adaptive architecture was more critical than hyperparameter tuning, with its design based on optimal performance in generating EMA data. As shown in Figure 2a, the generator mainly consists of two convolutional layers and two max-pooling layers, containing six and 31 neurons, respectively. The output of the final pooling layer is processed by a fully connected layer, followed by a softmax output layer. The convolutional layers use a kernel size of 5 with stride 1, while both the max-pooling layers employ a subsampling factor of (2, 2). The output layer is a fully connected layer with 401 neurons, matching the dimensionality of the input data prepared for the discriminator. Similar to WGAN-GP, the generator loss function in EMAGAN is expressed as

(7) $$L_{G}=-\frac{1}{m}\sum_{i=1}^{m}D\left(G\left(z\right)\right)$$

where *m* denotes the batch number. LeakyReLU is selected as the activation function for the generator. The discriminator shares a similar core architecture with the generator, as illustrated in Figure 2b. Major distinctions include the addition of a dropout layer (rate = 0.5) before the first convolutional layer to prevent network overfitting. Furthermore, the fully connected layer is reduced to 200 neurons, and the output layer contains a single neuron. The loss function of the discriminator is derived by taking the absolute value of Equation (6) and is formulated as follows:

(8) $$L_{D}=\left|-\frac{1}{m}\sum_{i=1}^{m}D\left(x\right)+\frac{1}{m}\sum_{i=1}^{m}D\left(G\left(z\right)\right)+\lambda \cdot gp\right|$$

where $gp={\left({\left\|\nabla_{\hat{x}}D\left(\hat{x}\right)\right\|}_{2}-1\right)}^{2}$, λ=0.2. During training of EMAGAN, EMA data from each operational scenario is individually fed into the network to learn its feature and subsequently generate corresponding simulated conductance data. The optimizers for both the generator and discriminator are based on the adaptive estimation method. Following parameter tuning, the learning rate was set to 0.0005, as determined through trial-and-error optimization. Here, the learning rate is a crucial parameter in the gradient descent algorithm, which determines the magnitude of parameter updates in each iteration of the GAN model. In GANs, it directly affects the training speed and convergence stability of the generator and discriminator. The total number of trainable parameters of the proposed EMAGAN model are 441,533 for the generator model and 320,933 for the discriminator model, and the FLOP for running the model is almost 80 TFLOPS. The next subsection gives a flow diagram for automated stress identification based on EMAGAN.

### 3.2. Flow Diagram for Enhanced EMA Data-Based Stress Identification

**Figure 3:**
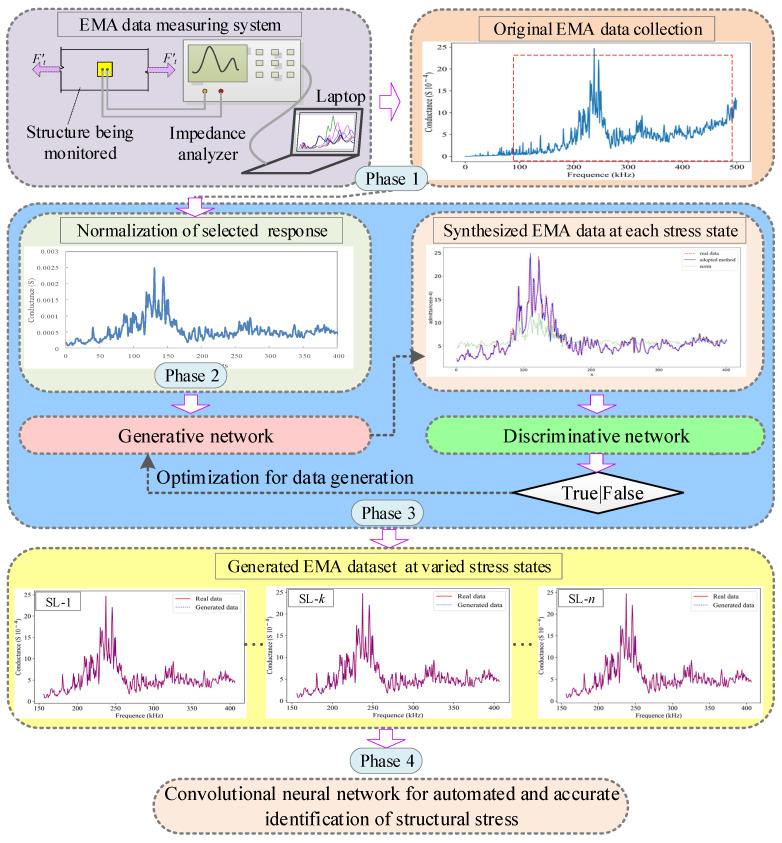
Flow diagram of the proposed approach.

Based on the EMAGAN, Figure 3 outlines the flow diagram for the automated stress identification approach. The process consists of four key phases: (i) constructing an EMA dataset using a specific PZT transducer on the target structure under varying tensile stresses; (ii) normalizing the critical sub-frequency responses to serve as input for the EMAGAN; (iii) generating data for each state through iterative training and testing of the EMAGAN model to determine its optimal hyperparameters; and (iv) feeding the hybrid EMA dataset into a CNN model to estimate the stress level. The following provides detailed explanations for each phase within the proposed framework.

Phase 1: Establishing the EMA dataset. To ensure the accuracy of the synthetic dataset, generally, hundreds of groups of data need to be collected for GAN training, for example, 1875 training and 469 acceleration data [74], for a total of 3900 samples in [75]. In this work, only 20 groups of EMA samples at initial unload and each stress state were obtained by the measuring system shown in Figure 1, which attempted to meet the reconstruction requirement for high accuracy. As previously noted, each EMA spectrum is typically sampled at 801 points during a single frequency scan. To optimize computational efficiency, this number is rationally reduced to 401 by focusing on the stress-sensitive response data. Once the EMA data is collected for each stress level and assigned corresponding labels, it undergoes data normalization prior to being fed into the EMAGAN, as detailed in the following phase.

Phase 2: Normalizing the EMA spectrums. At this stage, a novel data normalization algorithm is proposed to enhance the capability in learning and generating EMA data, based on the following reasons: (i) given that the actual errors between the EMA spectra induced by stress variation are relatively minor, the errors between the generated and original responses should be smaller than the actual ones between each state to ensure the validity of the generated data; (ii) a total of four steps are conducted to amplify the differences between the original signals. Step 1: The EMA data arrays $X_{i}^{n}$(*i* = 1, 2, …, 401) from *n* stress states are collected and fused as dataset $X\in R_{d}^{m\times n}$ (in the present study, *m* = 20, *n* = 6). Here, R represents the finite EMA set that contains all the EMA spectrums collected in the experimental tests on the aluminum beam under the axial tensile test. Step 2: Mean values $R_{d}^{m\times n}$ in the column direction were computed to achieve two vectors $R_{Avg}$ and $R_{Std}$ with a length of 401. Step 3: Each element in the row of $R_{d}^{m\times n}$ was subtracted via $R_{Avg}$ and divided by $R_{Std}$. Step 4: The datasets were input into the EMAGAN to produce the simulated datasets, and each element in every row of the new vector was added by 1 and finally multiplied with $R_{Avg}$. By applying this method, intrinsic similarities within the EMA signals were minimized, thereby accentuating subtle variations between samples. This amplification of discriminative features enhances their accessibility to the EMAGAN during the learning process. There are no other spatial correlations in the raw signals/material data that are handled, and the input samples are assumed to be independent and identically distributed before they are input into the later models.

Phase 3: Following the normalized original EMA signals, the data were fed into the model. The proposed normalized algorithm substantially enhances the performance of the GAN generator. To ensure that the capability of the GAN discriminator surpasses the output quality of the generator, its training frequency is four times that of the generator. Specifically, the discriminator undergoes four parameter updates for each single update of the generator. The generated EMA signals enhance the dataset for later CNN-based stress quantification; a total of 50 synthetic EMA sets were generated for one stress level (SL-0, … *k* …, *n*, here *n* = 5) and suitable for stress identification.

**Figure 4:**
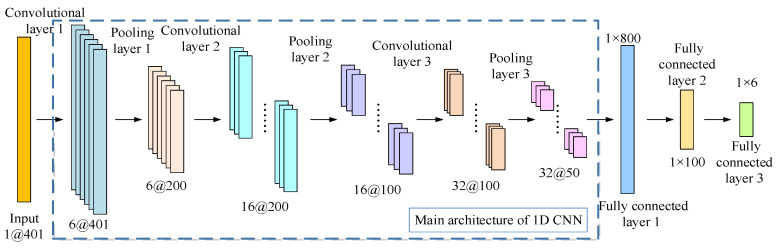
Architecture of the 1D CNN using raw conductance data as input.

Phase 4: 1DCNN for tensile stress quantification. The 1DCNN architecture, inspired by LeNet-5 [84], is tuned to integrate the featured EMA data with stress quantification stages, as illustrated in Figure 4. Adaptive hyperparameters for the 1DCNN model are configured to handle the input for the classification problem. The measured raw EMA spectrum with a size of 1 × 801 was reduced to 1 × 401 via selecting the critical sub-response to feed the CNN model. The kernel number for the convolutional layer is 5, the kernel size is 5 × 1 and the stride is 2, which are set to raise the efficiency of the CNN training. Considering three convolutional layers are utilized, the kernel size of the max-pooling layer is set as 2 × 1 with a stride of 2 to sufficiently subsample the input information. The total neurons of the three FCLs are respectively 800, 100 and 6 according to the stress levels being classified in the next section. Conductance arrays designated as training or testing datasets were assigned corresponding labels using one-hot encoding. To validate the efficacy of the augmented data for stress identification, the CNN model was trained and evaluated separately using only the original dataset and subsequently with the enhanced dataset, with their respective prediction results compared for analysis. Testing of the model trained by the hybrid dataset was also attempted using original data only. With the preceding work accomplished, then the prediction results of structural stress level could be output immediately. The next section covers the experimental validation of the approach.

## 4. Experimental Investigations

### 4.1. Experimental Procedure

**Figure 5:**
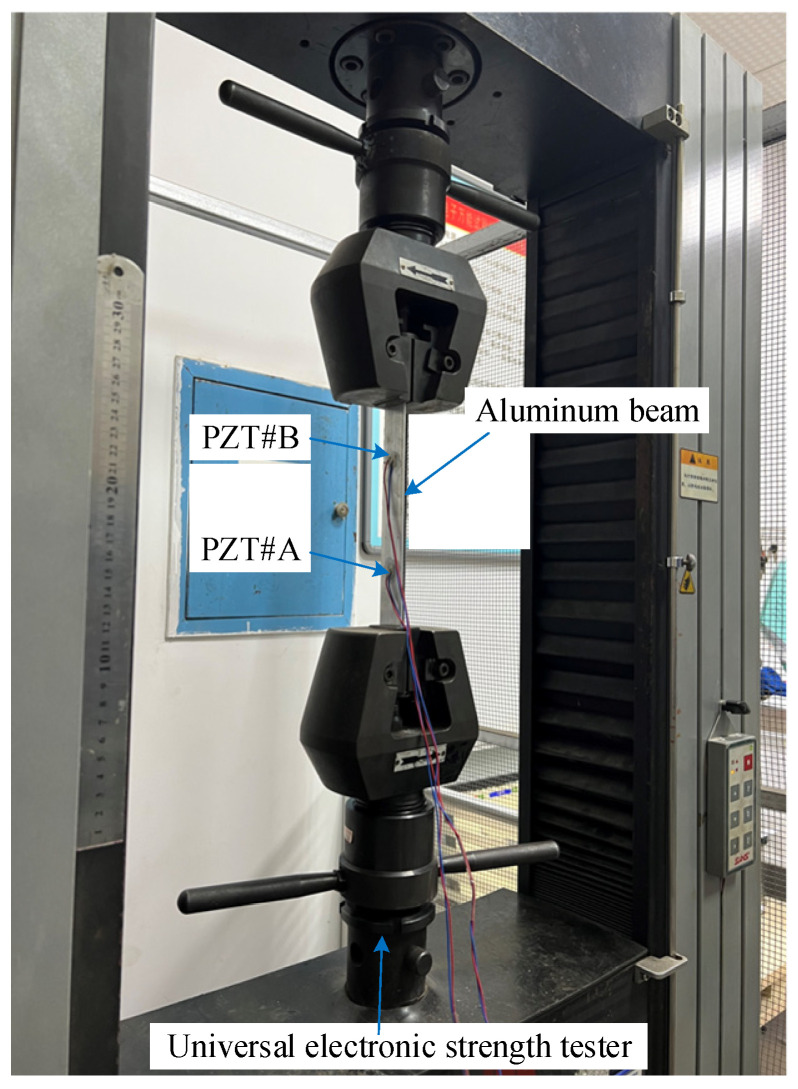
Experimental configuration for measuring the EMA signatures from an aluminum beam under an axial tensile test.

**Figure 6:**
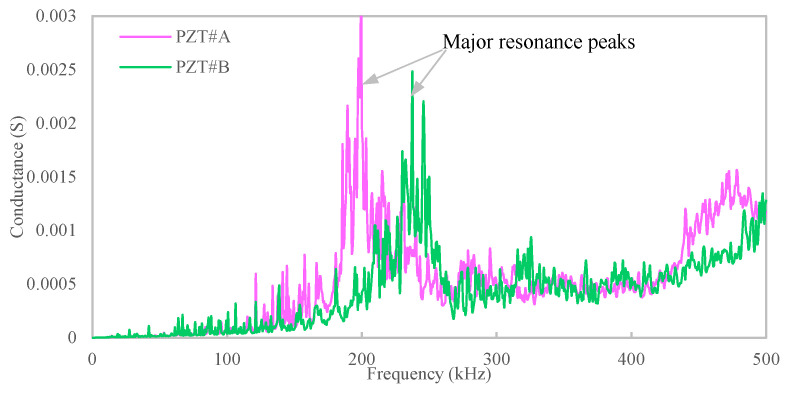
Conductance signature of the transducers bonded on the specimen before loading.

This part presents the validation of the proposed methodology, covering the EMAGAN-based generation of EMA data and the automated stress identification. In the experiment, two lab-scaled aluminum beam specimens with sizes of 300 mm × 30.3 mm × 2.2 mm were prepared for the tensile test. One was used for the strength test and the other was prepared for stress monitoring, as shown in Figure 5. Two PZT-5 patches, namely PZT#A and #B in dimensions of 10 mm × 10 mm × 0.5 mm, were bonded onto the same surface of the monitoring specimen for EMA data acquisition; their key properties are summarized in Table 1. Then, the two PZT patches were bonded at three equal points along the length of the specimen using an epoxy adhesive layer, which were 100 mm and 200 mm apart from the end, respectively. The adhesive layer was controlled with a thickness of approximately 0.1 mm through tightly pressing the patch to reduce redundant epoxy adhesive. The physical properties of the epoxy are the same as those in [42]. After allowing several days for the adhesive to fully cure, the specimen was ready for loading tests. A uniaxial tensile load was applied to both ends of the beam using a universal electronic strength tester, as shown in Figure 5. EMA signatures were recorded throughout the loading sequence, from the unloaded state through initial loading up to final fracture, under incrementally applied load steps. In the loading protocol, the tensile load was increased monotonically from a lower to a higher level at a constant rate of 100 N·s^−1^ until fatal failure. The strength of the prior specimen was tested as 225 MPa, and accordingly, six loading cases were considered for the latter as Case#0–#5, 0 kN, 3 kN, 6 kN, 9 kN, and 12 kN, and failure at 15 kN as Case#5. Correspondingly, the tensile stresses applied on the specimen were 0.00 MPa, 45.00 MPa, 90.10 MPa, 135.01 MPa, 180.02 MPa and 225.02 MPa. Detailed information and their prediction output labels for deep learning are tabulated in Table 2. It is worth mentioning that the numerical values reported with a precision of one to four decimal places in the later sections are mainly attributed to that some are determined by the supplier, some are caused by the uncertainty of the machine in the experimental tests. Under each loading step, the EMA signatures of both the PZT transducers are continuously measured in 20 groups by keeping the load applied on the specimen. The measuring system for the EMA signature consists of a laptop connected to a commercially available impedance analyzer. Voltage excitation on the PZT patches is 1 V. Through an Agilent Connection Expert, a local area network generated by the impedance analyzer is shared to the laptop for data collection and storage. Scanning frequency in each test ranged from 40 Hz to 500 kHz, with 801 discrete sample points. Conductance signatures of the transducers bonded on the specimen are shown in Figure 6. It is clear that the prominent resonance peaks of the two PZT patches are differently located at 200 and 230 kHz. It is worth noting that to focus on health monitoring of the host structure, subsequent analysis of EMA characteristics will primarily utilize the main resonance peaks below 400 kHz [11,12,13]. Moreover, it is worth mentioning that the tensile tests and data measurements are accomplished in a constant-temperature indoor environment; therefore, the temperature influence on the test results is reasonably avoided, which is critical in the continuous monitoring of practical metal infrastructures [85].

### 4.2. Stress Detection via Raw EMA Signature

**Figure 7:**
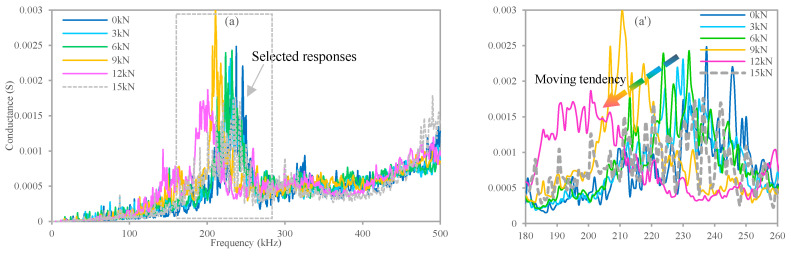

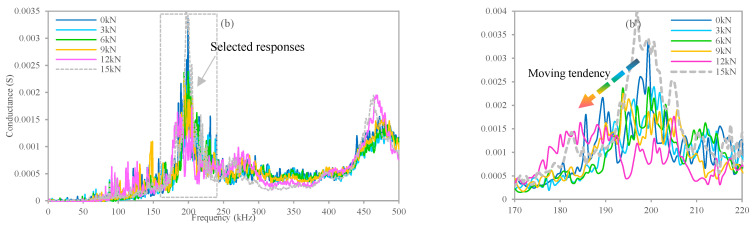
The 40 Hz–500 kHz band of Conductance spectra of PZT#A in band of (**a**) 40 Hz–500 kHz, (**a’**) 180-260 kHz and PZT#B in band of (**b**) 40 Hz–500 kHz, (**b’**) 170-220 kHz for the aluminum beam specimen under different loading conditions.

Firstly, qualitative detection of tensile stress is manually analyzed depending on the characteristics of raw EMA signatures. Since the conductance (real part) is more reactive than the susceptance (imaginary part) to damage or changes in structural integrity as well as the stress variations [14,40,41], conductance is primarily employed for stress identification in the latter analysis. Figure 7 shows the whole and selected frequency bands of raw conductance signatures for the aluminum beam specimen under different tension states. Obviously, for both PZT transducers, despite the intensive resonance peak clusters, the predominant band of the conductance spectrum including the whole and zoomed bands of 170–270 kHz and 170–230 kHz shows uniform leftward and downward (LD) shifts with the increment of load steps up to 12 kN. These features could be attributed to the enlargement of point-wise dynamic stiffness as well as the driven-point mechanical impedance caused by tensile stress, which are in good agreement with the analytical and experimental results of the tension/compression stress effect on the EMA signatures of RC structures or aluminum beams subjected to uniaxial tensile load [18,41]. However, at the failure load of 15 kN, the conductance spectra for both PZT transducers behave conversely rightward and upward (RU), shifting back to the initial unloading state; this is because at this state, the tested specimen has been separately snapped, and there is no more tensile stress imposed on the specimen as well as the PZT transducers. It is worth mentioning that according to sensor diagnostics in the EMA technique, there is no sensor fault or debonding defect occurring during the test, as a sensor fault would result in the whole band of signal loss, while debonding causes uniform downward shifts with magnitude reduction in all resonance peaks of the EMA spectrum [86,87]. Therefore, regular features of the EMA signature demonstrate its effectiveness for the evaluation of tensile stress applied on the beam, which makes it feasible to quantify the stress level via using deep learning of these features.

**Figure 8:**
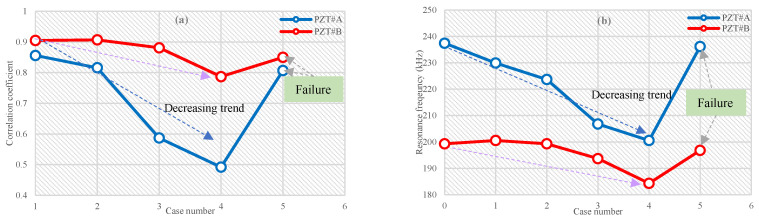
(**a**) CC and (**b**) resonance frequency of conductance spectra for PZT#A and PZT#B under different loading conditions.

As seen from Figure 7, it is clear that the 150–400 kHz frequency responses contain the majority of resonance peaks between the PZT and the aluminum beam specimen, which also shows the most dramatic changes when subjected to different tensile stresses. Therefore, this band of frequency responses is selected as the feature representations for the latter deep learning-based stress detection. The resonance feature representations indicate the interrogation between the PZT and the structures being monitored, and the variations in these resonance peaks hold the key to understanding the structural property changes. Structures made from metal materials such as steel, aluminum and their composites are active and have the highest efficiency for EMI applications, as they result in more and intense interactions between the structure and PZT. Conventionally, to quantify the absolute changes in the EMA signatures responding to stress variations, a statistical indicator, namely CC, in addition to the shift in the maximum resonance frequency, are computed and comparatively depicted, as shown in Figure 8. Taking the unloading state as reference, the CC values decrease from 0.856 to 0.492 for PZT#A and from 0.905 to 0.787 for PZT#B with the stress development before fracture, respectively. To some extent, a reduction in the CC values indicates that the absolute deviations between conductance signatures are gradually amplified by stress growth; this result coincides with the previous studies [26,27]. Similarly, maximum resonance peaks of the conductance spectra decrease from 237.44 kHz to 200.57 kHz for PZT#A and from 199.32 kHz to 184.32 kHz for PZT#B. At a fracture state, CC values recover to 0.807 and 0.850 and a maximum resonant frequency of 236.19 kHz and 196.82 kHz for the two transducers, respectively, due to the fact that no stress is regained on the specimen when it is totally broken. More specifically, corresponding to the stress increment from 0% to 20%, 40%, 60% and 80% of the ultimate tensile strength of the specimen, the shifts in the maximum resonance frequency decrease by 0.63%, 0.00%, 2.82%, and 5.52% and 0.22%, 2.65%, 2.82%, and 7.53% for the two PZT transducers, and the CC index values decrease by 4.67%, 31.43%, and 42.52% and 3.16%, 5.79%, and 13.04%, respectively. Nonetheless, it is difficult to give accurate information about the stress level applied on the specimen merely using the EMA signatures and their derived indicators. This issue is expected to be solved by using a deep learning approach in a later section.

### 4.3. Enhancement of EMA Signals

**Figure 9:**
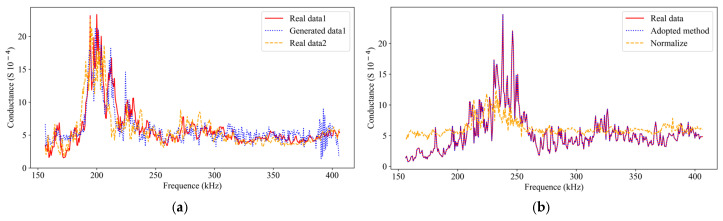
Comparative results of generated conductance spectrum based on (**a**) unnormalized and (**b**) normalized data using the proposed and traditional methods.

This section details the enhancement for the EMA signatures via the EMAGAN framework. As previously indicated, the efficacy of the produced data is established when its deviation from the original data under identical working conditions is less than the variation observed between original datasets from different conditions. To this end, the generation effect is first testified via using the EMA data processed by different methods, including an unnormalized, traditional *z*-score algorithm and the proposed normalized approach. During the running process of the EMAGAN model, the random seeds are fixed despite the initial parameters being randomly produced, and fixing the random seeds is mainly intended to ensure the repeatability of the reported results. The variances across multiple runs are all lower than 0.05, and this result can also ensure the repeatability of the reported results. The EMAGAN was trained over 1000 epochs to evaluate its performance. Results show that around the 780th epoch, the absolute loss fluctuated and decreased from approximately 0.3 before converging to a stable value near 0.05. On average, generating 50 groups of EMA signatures using the EMAGAN required only 15.48 s per case. Compared to the traditional measurement method, which takes over 2 min to manually capture a single EMA signature, the EMAGAN demonstrates a speed increase of more than 380 times, substantially reducing the time required for data collection. Figure 9a compares the generated conductance spectrums between 250 and 650 sample points for Case#1. Using the unnormalized original data and using the real data in Case#1 and Case#2, it is seen that the generated data is poor quality, as there are too many oscillations nearby the original sample points. Mean square error between the real and generated data is 2.36 × 10^−4^, which is even larger than 2.17 × 10^−4^ of that between the real data in Case#1 and Case#2. Additionally, as shown in Figure 9b, the generated data using the traditional *z*-score normalized method tagged as “Normalize” is greatly discrepant with the real one, while the generated conductance perfectly matches with the original one when using the proposed method, of which the error is merely 1.05 × 10^−5^. It is worth mentioning that in this study, the data normalization and preprocessing steps are applied globally for the 20 sets of the EMA data under the same load condition recorded as one group, and they are processed in batches instead of independently per sample as traditional normalized methods do. Such a preprocessing method limits the size of the input data to be around 0 and ensures that it becomes neither too large nor too small. Therefore, no further normalization is required for the data. The difference between this method and traditional normalization lies in that traditional normalization performs normalization on each piece of data separately. For the data used in this paper, which is in the column direction, the mean and standard deviation are calculated, which are the mean and standard deviation of each 801-dimensional data. This can also limit the data within a certain range around 0. Therefore, the preprocessing method used in this paper not only significantly reduces the similarity between different data but also increases the difference, making the network learning faster. The results shown in Figure 9 demonstrate that this method significantly improves the generation effect. These results demonstrate that data normalization profitably reduces more than 22.5 times of errors for the generated data. The proposed normalization approach efficiently overcomes the limitation of traditional ones for EMA data generation, and thus could be synergistically employed with the EMAGAN for data synthesis in practical application.

**Figure 10:**
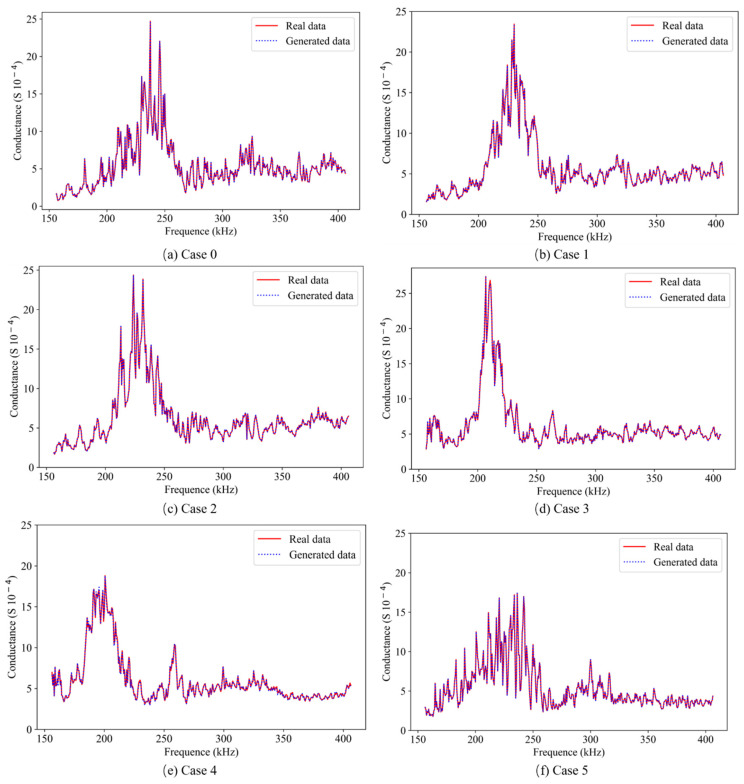
Original/generated EMA signals of PZT#A at (**a**) Case#0, (**b**) Case#1, (**c**) Case#2, (**d**) Case#3, (**e**) Case#4, (**f**) Case#5.

**Figure 11:**
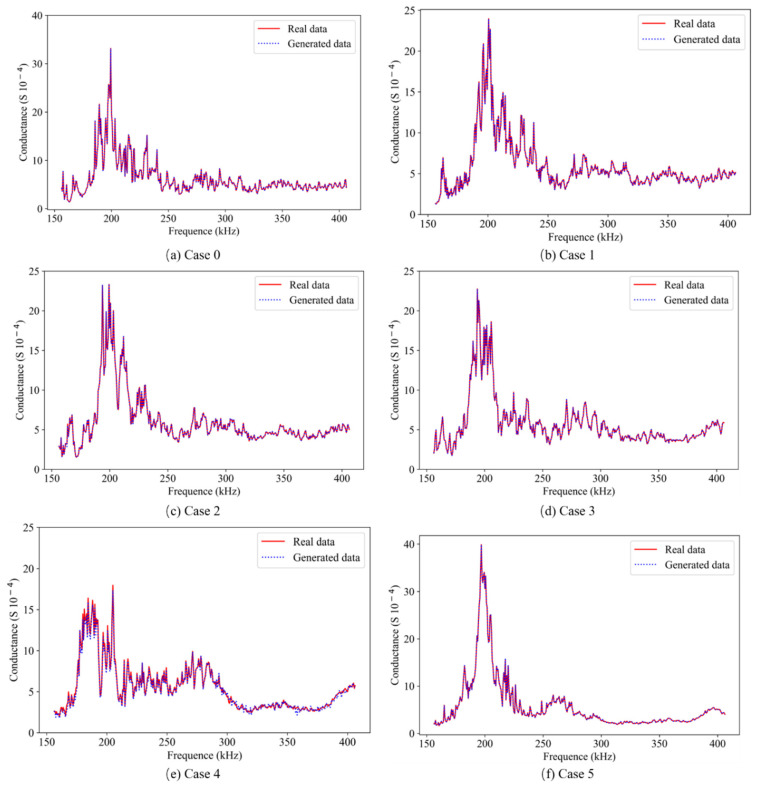
Original/generated EMA signals for PZT#A at (**a**) Case#0, (**b**) Case#1, (**c**) Case#2, (**d**) Case#3, (**e**) Case#4, (**f**) Case#5.

**Figure 12:**
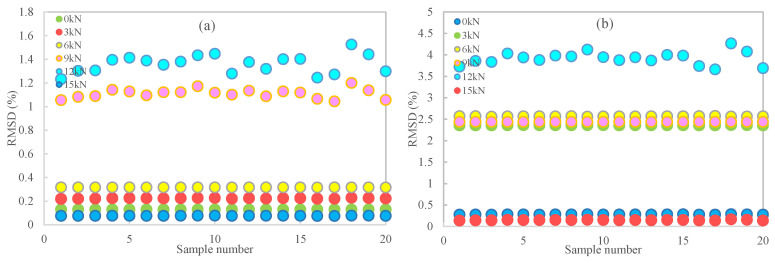
RMSD of the generated and original EMA samples for (**a**) PZT#A, (**b**) PZT#B under different loading conditions.

**Figure 13:**
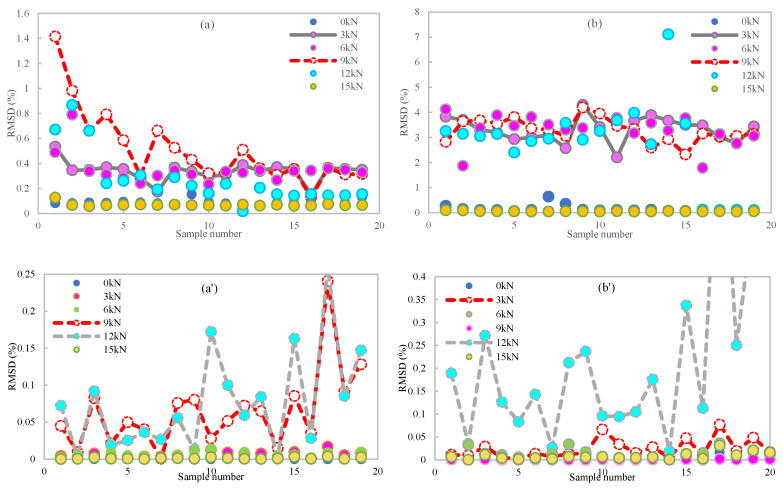
RMSD of conductance signatures between each other for (**a**) original, (**a′**) generated data of PZT#A, and (**b**) original, (**b′**) generated data of PZT#B.

Figure 10 presents a comparison between the original and EMAGAN-generated conductance signals from PZT#A under different tensile conditions. The results demonstrate that the generated spectra align closely with the original ones, accurately replicating both the resonant peaks and the diverse curve shapes. Figure 11 also shows similarly favorable results for PZT#B. Except for the high consistency between the synthetic and real data, it is found that the differences at a loading state of 12 kN are relatively large to be visually discriminated from the comparative spectra for both PZT transducers. Such a phenomenon is possibly attributed to the substantial deviations that existed in the original dataset caused by the high-level stress effect on the original EMA signatures of the beam transformed from an elasticity to plasticity stage, as depicted in Figure 8. The larger RMSDs at a load of 12 kN indicate more dramatic variations in the EMA signatures, including the larger LD shifts in the resonant peaks and frequencies that are caused by the physical changes at an unstable plastic state, particularly the significant stiffness reduction and damping increment of the aluminum beam specimen according to the character diagnostics of the EMA technique [85,86]. To verify the accuracy and evaluate the errors of the generated data, two aspects of comparative investigations are accordingly performed. On one hand, RMSD values between the generated and original conductance samples are calculated, as shown in Figure 12. It is seen that there is a little change in the index values for each stress state, all that are smaller than 1.8% and 4.5% for PZT#A and #B, respectively. With the growth in tensile force from 0 kN to 12 kN, the higher the stress level, the larger the index values, while at the load states of 0 kN and 15 kN, the deviations are all below 0.5%. On the other hand, the RMSD of each conductance sample for the original and generated dataset is computed and shown in Figure 13. It is observed that for both PZT transducers, the index values of the original samples are significantly higher than those of the generated ones. The majority of the original RMSD values are less than 5%, and most of the generated ones are even 1%. The RMSDs for the generated conductance signatures at a load of 12 kN show more significant fluctuations and absolute values than the other loading conditions, which indicates that plastically, physical changes impose an influence on the original EMA signatures as well as the generated ones, even though the deviations are mostly less than 0.4%. These observations further confirm the reliability of the EMAGAN for generating EMA signatures under dramatic conditions. Moreover, the errors for the original/generated data at initial unloading (0 kN) and post-failure (15 kN) states are much smaller than those at loading conditions, which is in accordance with the results in Figure 12. This result, to some extent, demonstrates that the high-level stress state that influenced the original EMA signature of the specimen has a corresponding impact on the generated signatures. Such an impact is insignificant on the accuracy of the generated EMA data. In summary, the EMAGAN can generate required EMA signals with high quality and efficiency to assist the deep learning-based stress quantification, as depicted in the next section.

### 4.4. Stress Quantification via Enhanced EMA Signature

**Figure 14:**
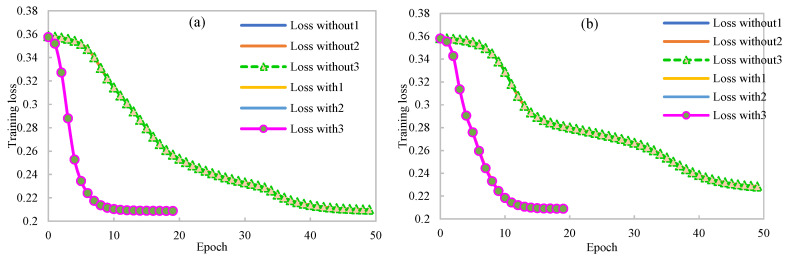
Training loss of the CNN model with and without data enhancement for (**a**) PZT#A and (**b**) PZT#B.2.

**Figure 15:**
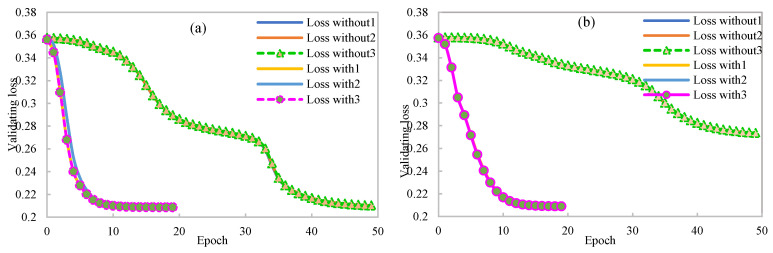
Validating loss of the CNN model with and without data enhancement for (**a**) PZT#A and (**b**) PZT#B.

**Figure 16:**
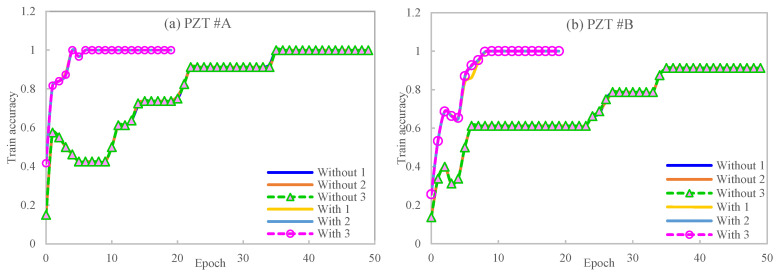
Training accuracy for 1DCNN by employing the conductance signals with and without data enhancement for (**a**) PZT#A and (**b**) PZT#B.

**Figure 17:**
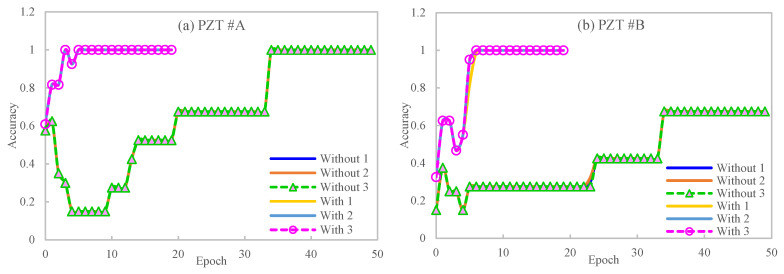
Accuracy for validating the 1DCNN model by employing the conductance signatures with and without data enhancement for (**a**) PZT#A and (**b**) PZT#B.

This section evaluates the stress quantification using an enhanced EMA dataset. As outlined in Table 3, three distinct datasets were constructed to train, validate, and test the effectiveness of the synthetically generated EMA signals. Firstly, the efficiency and accuracy were compared between the enhanced dataset (hybrid dataset #1 in Table 3) and the non-enhanced original dataset (real dataset #2 in Table 3). Network training was conducted using the respective training sets, with the remaining samples reserved for validation. To minimize randomness from factors such as network initialization, each model was trained three times under identical conditions. Figure 14 and Figure 15 illustrate the training and validating loss of the CNN models with and without EMA data enhancement. It can be seen that the models can rapidly converge after a certain amount of training and validating epochs. Generally, the models trained and validated by the data without data enhancement require more iterations, i.e., 40 epochs when compared to that by the enhanced datasets merely requiring 10 epochs, which indicates the elevated generalization performance of the data enhancement and good stability of the models. As for the training and validating accuracies, Figure 16 and Figure 17 show the prediction accuracy during training and validation for models using enhanced versus non-enhanced data. During training with the non-enhanced dataset, accuracy improved gradually with intermittent plateaus. For PZT#A, 100% accuracy was attained after 36 epochs, whereas PZT#B reached only 91.25% at convergence. As for the one trained by the enhanced dataset, there was a rapid increase to reach a 100% prediction accuracy after only seven and 10 epochs of iteration for PZT#A and #B, respectively. Similar trends were observed during model validation, as shown in Figure 15. Using enhanced data, validation accuracy rose rapidly to 100% within six epochs for PZT#A and eight epochs for PZT#B. However, due to that only utilizing the original dataset requires more than 35 and 50 epochs to convergence, prediction accuracy could reach a hit rate of 100% and only 67.50% for PZT#A and #B, respectively. The lower validation accuracy for PZT#B is attributed to overfitting when training solely on the original data. These findings demonstrate that data enhancement via EMAGAN not only improves identification accuracy and accelerates convergence but also helps mitigate overfitting in CNN-based stress quantification models.

**Figure 18:**
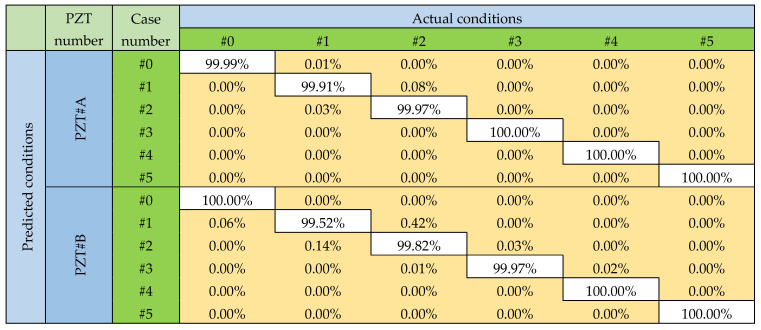
Classification probability of the predicted stress level by CNN.

Secondly, a cross test of the CNN was additionally performed via feeding the real data to the model that was trained by the hybrid datasets (i.e., no. #3 dataset in Table 3), not only to prove the effectiveness of the generated dataset00000 but also to give a little insight into the prediction accuracy of the CNN model. As illustrated in Figure 18, the average classification probabilities for each predicted stress state are presented. The prediction probabilities consistently exceed 99.52%, with the majority reaching 100%, while false identification rates remain below 0.5%. Here, the identification rates are the proportion of the total samples that the model correctly identifies as belonging to the target category. Minor prediction variability is observed primarily for PZT#B, likely attributable to irregularities in its original EMA signatures, as shown in Figure 7. The high overall accuracy reflects a strong agreement between the predicted and actual stress levels. These results affirm the high quality of the generated EMA datasets, demonstrating their suitability as effective substitutes for real measurements in deep learning-based tensile stress quantification. The proposed model not only ensures rapid convergence but meanwhile maintains high identification accuracies. Different than traditional EMA techniques for stress and damage detection via artificial analysis on the characteristics of the EMA signatures including the resonant peaks and resonant frequencies, they are qualitative in essence. It is difficult to estimate the stress level, and moreover they are overly dependent on empirical knowledge or theoretical impedance models. The uniqueness of the solution presented here provides an accurate stress level on the metal materials or structures once the model is trained by high-quality EMA signals correlated to certain stress. This approach enables accurate prediction of stress levels in aluminum beam specimens under subsequently applied loads. The presented solution preferably eliminates the laborious analysis and further enhances the practical application potential of the EMA technique in the metal SHM field. Therefore, the EMAGAN collaborated with the CNN approach makes it promising for in situ stress monitoring of in-service metallic structures, particularly encountering data deficiency in practice.

## 5. Conclusions

This paper presents a novel EMA enhancement framework based on EMAGAN to produce sufficient EMA datasets for the quantification of metal structural tensile stress using deep learning models. The framework is validated through a uniaxial tension experiment on an aluminum beam specimen. It eliminates the inefficiency and difficulty of continuously measuring EMA signals under stress-varied scenarios and promotes the automated identification of the structural stress level. From the results, the following conclusions can be drawn:

(1) Qualitative identification of axial tensile stress applied in an aluminum beam specimen shows that resonance peak clusters of the conductance spectrums for two bonded PZT transducers have uniform LD shifts (i.e., decrease both in the resonance frequency and magnitude) with the increment in load steps before failure. Corresponding to the stress increments of 20%, 40%, 60% and 80% of the ultimate tensile strength, quantifiable variations in the maximum resonance frequency shift, decreasing by 0.63%, 0.00%, 2.82%, and 5.52% and 0.22%, 2.65%, 2.82%, and 7.53%, while those of the CC index values decrease 4.67%, 31.43%, and 42.52% and 3.16%, 5.79%, and 13.04% for PZT#A and #B, respectively. The random relationship between stress and EMA variations makes it difficult to accurately identify the stress level.
(2) The validity test of the EMAGAN demonstrates that the pre-normalization of original EMA signals affects the accuracy of synthetic ones, which collaborated with a new normalized algorithm that has errors less than 22.5 times compared to using traditional ones. Training of the EMAGAN was conducted to generate 50 groups of eligible conductance spectra that perfectly matched the original ones regardless of intensive resonance peak clusters, merely costing approximate 15 s for each case and exceeding 380 times faster than the normal measurement method. Performance evaluation shows that all errors that existed in the RMSD index between the generated and original conductance datasets are respectively less than 1.8% and 4.5% for the two transducers, while those between the generated ones are merely around 1%.
(3) Enhanced EMA datasets feeding to an adaptive CNN model for the quantification of tensile stress demonstrate that the training and validation accuracy for PZT#A and #B could reach to 100% in a few seconds when using the enhanced dataset, which is superiorly higher than that without data enhancement. A cross test of the CNN model by feeding the real datasets to the model trained by generated ones indicates that prediction probabilities are all over 99.52% and most even up to 100%. Results confirm that data enhancement improves identification accuracy, overcomes overfitting and accelerates convergence of the CNN model, which consequently provides a possible paradigm of data-driven stress identification of in situ metal structures.

It is worth emphasizing that, while the proposed EMAGAN can generate qualified EMA signatures by learning their feature distributions, this study represents a very preliminary study of applying GANs to EMA signal generation for metal structures. Despite that considerable efforts have been made to modify the baseline model network, such as that it solves the gradient disappearance in the low-dimensional manifold as well as removes the sigmoid activation function at the last layer of the discriminator and sets a threshold value to the output layer parameter of the discriminator, a more rigorous comparison would require aligning model capacity, including the loss function, the amount of convolutional layers, the kernel sizes, the neurons in the fully connected layers and addition of a dropout layer, and so forth. These are still required for deriving more explainable networks in future investigations. Limitations remain and should be addressed in future work. First, the current validation is limited to EMA data from aluminum structures under uniaxial tensile loading. Further experiments on other in-service structures, such as concrete or composite materials, and under complex stress or damage conditions, are necessary to determine the broader applicability of EMAGAN. Second, only one CNN architecture has been evaluated in this study. Systematic hyperparameter investigations and comparative studies with other GAN-based methods are needed to optimize the framework. Third, beyond stress classification, EMA-based SHM also faces challenges of data scarcity and imbalance in tasks such as damage detection and localization, which warrant further in-depth research. Fourth, the temperature effect on the tensile stress detection as well as the thermally disturbed EMA data have not been investigated in this study; further investigations on the effectiveness of the EMAGAN for handling the thermally disturbed EMA data should be systematically investigated, as temperature possibly drifts the EMA spectrums more overwhelmingly and significantly than that caused by the tensile stress. Fifth, the authors merely examine the tuned EMAGAN with the majority protype of the WGAN-GP including the loss functions, and more systematic investigations about the models with different loss functions such as focal loss, class-weighted loss, or margin-based losses should be tried in future works, particularly for dealing with class imbalance issues. Sixth, despite the preferable results, more studies on the robustness of the model to sensor noise, measurement drift caused by ambient temperature, or missing data recovery should be conducted in future works. These issues, in addition to the controlled perturbation experiments, would help assess stability under realistic operating conditions. These realistic operating conditions are also guaranteed to enhance the reliability of the proposed approach. Seventh, the analysis of the feature importance or internal representations of the deep learning tasks, including those using attention weights, saliency maps, or sensitivity analysis, would definitely help verify that the model is learning physically meaningful patterns rather than spurious correlations. The knowledge about these internal physical analyses requires more deep investigations on explainable neural networks. Finally, although EMAGAN requires only around 20 samples for training, reducing this number remains desirable. Future work will explore training the model with only one or two samples to further enhance its data efficiency.

## Figures and Tables

**Table 1:** Properties of PZT.

Item	Density $\left(g/{cm}^{2}\right)$	Piezoelectric Coefficients $\left(C/N\right)$	Resistance $\left(m\Omega \right)$	Curie Temperature $\left(℃\right)$	Electromechanical Coefficient	Piezoelectric Coefficients (m/V)
PZT-5	7.86	≥400 × 10^−12^	≥1000	≥330	0.8	$\left[\begin{array}{ccc} 0 & 0 & -2.1\times 10^{-10}\\ 0 & 0 & -2.1\times 10^{-10}\\ 0 & 0 & 5.0\times 10^{-10}\\ 0 & 0 & 0\\ 0 & 5.8\times 10^{-10} & 0\\ 5.8\times 10^{-10} & 0 & 0 \end{array}\right]$

**Table 2:** Case labels and stress level in the loading test.

Case Label	Applied Load (kN)	Stress Level (MPa)	Prediction Output
#0	0	0.00	[1, 0, 0, 0, 0, 0]
#1	3	45.00	[0, 1, 0, 0, 0, 0]
#2	6	90.01	[0, 0, 1, 0, 0, 0]
#3	9	135.01	[0, 0, 0, 1, 0, 0]
#4	12	180.02	[0, 0, 0, 0, 1, 0]
#5	15	225.02	[0, 0, 0, 0, 0, 1]

**Table 3:** Data for training, validation and testing of the deep learning model for stress identification.

Data Number		Item	Data Component	Amount
#1	With enhancement	Training	Real + generated dataset	300
		Validation	Real + generated dataset	120
#2	Without enhancement	Training	Real dataset	80
		Validation	Real dataset	40
#3	Model test	Training	Real + generated dataset	300
		Testing	Real dataset	120

## Data Availability

The original contributions presented in this study are included in the article. Further inquiries can be directed to the corresponding author.
